# Investigation on uniaxial compression and fracture damage mode of prefabricated parallel double-jointed red sandstone

**DOI:** 10.1371/journal.pone.0305565

**Published:** 2024-06-17

**Authors:** Xiaojuan Xie, Zhanxin Liu, Kun Ding, Yajun Xiao, Zhengqi Zhang

**Affiliations:** Shaanxi Railway Institute, Shaanxi Weinan, China; Sapienza University of Rome: Universita degli Studi di Roma La Sapienza, ITALY

## Abstract

As a special type of joint fracture, the fracture evolution characteristics of parallel double joints have important engineering significance for the stability analysis of fractured rock mass. In this work, a new method for calculating stress intensity factor of parallel double-jointed fractures was importantly proposed. Physical uniaxial compression tests were carried out on parallel double jointed red sandstone filled with cement mortar under different geometric parameters, and the macroscopic mechanical properties and failure characteristics of red sandstone are deeply analyzed. The results show that the larger the connectivity rate is, the smaller the peak stress and strain are. The increase of connectivity rate will affect the change rate of transverse strain in the center of rock bridge. The closer the dip angle of the joint is, the lower the peak stress is and the shorter the failure time is. The damage mode of joint tip encroachment affects the lateral displacement of the rock bridge center, and the displacement is always close to the first damage section. The closer the joint tip is to the load, the easier the end-face penetrating cracks occur. The research content can provide basic support for guaranteeing the stability of underground engineering rock mass.

## Introduction

Fractured rock mass stability is a major research field in rock slope engineering, and its sudden and destructive nature directly threatens the safety of people’s life and property [1–4]. Under the action of geo-stress field, the geological structure forms micro-structural planes such as main joints, fractures and fault fracture zones, which make the rock mass structure present mechanical properties such as discontinuity, heterogeneity and anisotropy, and the overall strength of rock mass is reduced [5–9]. The excavation of rock mass in roadway engineering causes the disturbance of surrounding rock, which induces the expansion and evolution of a large number of primary joint planes in rock mass, and gradually breaks down and evolves into macroscopic structural planes. The existence of macroscopic structural plane greatly threatens the safety construction of underground engineering. Before analyzing the macroscopic mechanical properties and failure characteristics of jointed rock mass, it is necessary to study the fracture damage characteristics of microstructure surface under stress.

The main joints must have microstructure planes, which can be divided into penetrating joints, spreading joints, non-penetrating joints (discontinuous joints) and closed joints regarding the development degree of joints. When the strength and failure mode of rock mass are mainly affected by discontinuous joints, it is called non-persistent jointed rock mass [10]. The uniaxial compressive mechanical properties of rock mass have always been the basis and focus of the research on the mechanical properties of jointed rock mass [11–13]. Wei et al believed that there is a significant difference in the strength of rocks under uniaxial tension and compression, and they verified that the elastic modulus of rocks under compression is 2~4 times that under tension [14]. Fu et al studied the failure behavior and mechanism of fracturing in the gypsum specimens containing vertical notches under uniaxial compression and elucidated the dominant tensile failure behavior of the specimens with cracks [15]. Hu et al made a large number of two-layer rock-like samples with different material combinations and fracture angles of 30°, 45° and 60° respectively, and carried out a series of uniaxial compression tests [16]. According to Lemaitre strain equivalence hypothesis, Chen et al established an intrinsic damage model for discontinuous fractured rock mass with respect to Moore-Coulomb criterion, considering the coupling effect of macro and micro defects [17]. Li et al established a constitutive model for hysteresis shearing and associated energy dissipation of rock joints, and derived the analytical expressions of the model during cyclic shearing processes, which is more convenient in application [18]. Hoek and Bieniawski suspected that crack growth caused by a single crack could not fully explain the macroscopic fracture behavior of the specimen [19]. Chen et al investigated the scaling effect on the shear mechanical properties of non-through horizontal rock-like joints at different scales by performing a series of straight shear test numerical calculations [20]. Haeri et al proposed multilaminate-based model and presented to predict the strain hardening behavior of rock reproduce accurately the mechanical behavior of rocks [21]. Suebsuk et al discussed the plastic volumetric deformation of naturally structured clays before the initial yield, which can be used to simulate structural soils under compression and shear response in geological and geotechnical engineering problems [2]. Wang et al established different joint models, geometry joint models and complex fracture network physical models based on 3D printing technology[22]. Cao et al used RYL-600 shear rheometer to conduct several shear tests on artificial granite specimens under different normal stresses, and obtained shear stress-shear displacement curves under different normal stresses[23]. Shkuratnik et al conducted acoustic emission and strain measurements on rock salt samples to analyze the stability of salt rock and the change of parameters during the progressive creep stage [24]. Based on uniaxial compression test and rock energy principle, Wang et al established the crack extension and strength damage criterion based on the change of elastic energy consumption ratio for non-through jointed rock body [25]. Yang et al conducted uniaxial compression tests on fractured fine sandstone with different geometric states, and emphasized the effects of bridge angle and bridge width on crack initiation, extension process and damage mode of crushed fine sandstone specimens [26]. Lee and Jeon studied crack initiation, propagation and merging at or near open cracks or defects in specimens under uniaxial compression [27]. Suo et al conducted uniaxial compressive strength tests on core samples at different lamination angles (0°, 30°, 60° and 90°) to study the mechanical properties and failure modes of shale [28]. Zhao et al analyzed the crack evolution law of rock-like specimens with cracks in the whole loading process [29]. Some scholars believe that crack initiation threshold (such as crack initiation stress and crack damage stress) is also closely related to the energy evolution of rock [30–33]. In addition, Ivashenko et al conducted uniaxial compression and biaxial compression tests on concrete in variable load-strain mode and investigated concrete strain, internal damage accumulation and ultimate strain value changes [34]. Nitka et al characterized the behavior of plain concrete during uniaxial compression and uniaxial tension by using the discrete element method, and analyzed the effects of concrete density, aggregate size and specimen size on the stress-strain curves, volume changes and fracture processes[35]. Tien et al. conducted uniaxial compression experiments on simulated transverse isotropic rocks, and analyzed and classified their failure mechanism based on circumferential surface images of cylindrical specimens recorded by rotary scanners[36]. In summary, there are fewer studies on non-through jointed red sandstone, especially for red sandstone with filling fissures in parallel double joints. As a special type of joint fracture, the analysis of parallel double joints is of great significance in analyzing the fracture evolution characteristics for the macroscopic mechanical properties and damage characteristics of jointed rock bodies.

In order to further explore the mechanical properties and crack propagation law of non-persistent double-jointed rock mass under different geometric parameters, the red sandstone samples collected on site were prefabricated and cut in the laboratory, and the prefabricated cement mortar filling double-jointed fissure samples under different geometric parameters were subjected to uniaxial loading test. The effects of joint dip Angle, bridge dip angle and joint connectivity on rock mass strength and deformation characteristics under uniaxial compression were systematically studied. This paper mainly analyzes mechanical characteristics, stress-strain relationship and acoustic emission characteristics of red sandstone with parallel double joints under uniaxial action, and explores the anisotropy of failure modes and crack propagation law of rock bridge. It is hoped to provide some basic support for studying the engineering behaviors such as the expansion and evolution of joint fissure and the reinforcement of jointed rock mass, and evaluating the safety state of underground engineering rock mass and the stability of engineering structure.

## Damage constitutive model of rock mass with discontinuous joints

### Rock mass damage variables of double joints

According to the stress characteristics and failure forms of rock mass fractures, they are usually divided into three basic fracture types [37]: opening type (type I), sliding type (type II) and tearing type (type III). In geotechnical engineering, since the tensile strength of rock is usually much smaller than the compressive strength and shear strength, the fractures of rock are mainly I, II or I-II composite fractures. Based on the integral method and plane strain in fracture mechanics, the calculation formula of damage variable *D* is derived by using the principle that the change of elastic strain energy caused by elastic joints is equal to the increase of additional strain energy [38,39]:

$$D=1-\frac{1}{1+\frac{2}{V}\frac{\left(1-v^{2}\right)}{\sigma^{2}}\int_{0}^{A}\left(k_{I}^{2}+k_{\mathrm{II}}^{2}\right)dA}$$ (1)

Where, *V* is the volume of the object, *ν* is the Poisson’s ratio of the elastomer, *σ* is the uniaxial compression stress, *k*_*I*_ and *k*_*II*_ are the type *I* and *II* stress intensity factors at the joint tip respectively, and *A* is the joint surface area.

Under uniaxial stress state, elastic strain energy *U*^*E*^ can be calculated by [39]:

$$U^{E}=\frac{\sigma^{2}}{2E{\left(1-D\right)}^{2}}$$ (2)

Where, *E* is the elastic modulus of the elastomer.

It can be seen from the above formula that the key problem in calculating the damage variable of jointed rock mass is the calculation of stress intensity factor at the joint tip. Combined with the calculation method of stress intensity factor considering the influence of joint geometry, strength parameters and transverse stress of joint tip on stress intensity factor in previous publication [38]. Here, the influence of the geometrical shape and strength parameters of the non-interpenetrated double-layer joint on the stress intensity factor is studied, and the calculation method of the stress intensity factor at a certain point in the double-layer rock is proposed. The variable is introduced to modify the stress intensity factor, and the modified stress intensity factor is substituted into Eq (1) to obtain the corresponding calculation method of damage variable of non through double jointed rock mass.

### Stress intensity factor of parallel twin joint model

**Fig 1:**
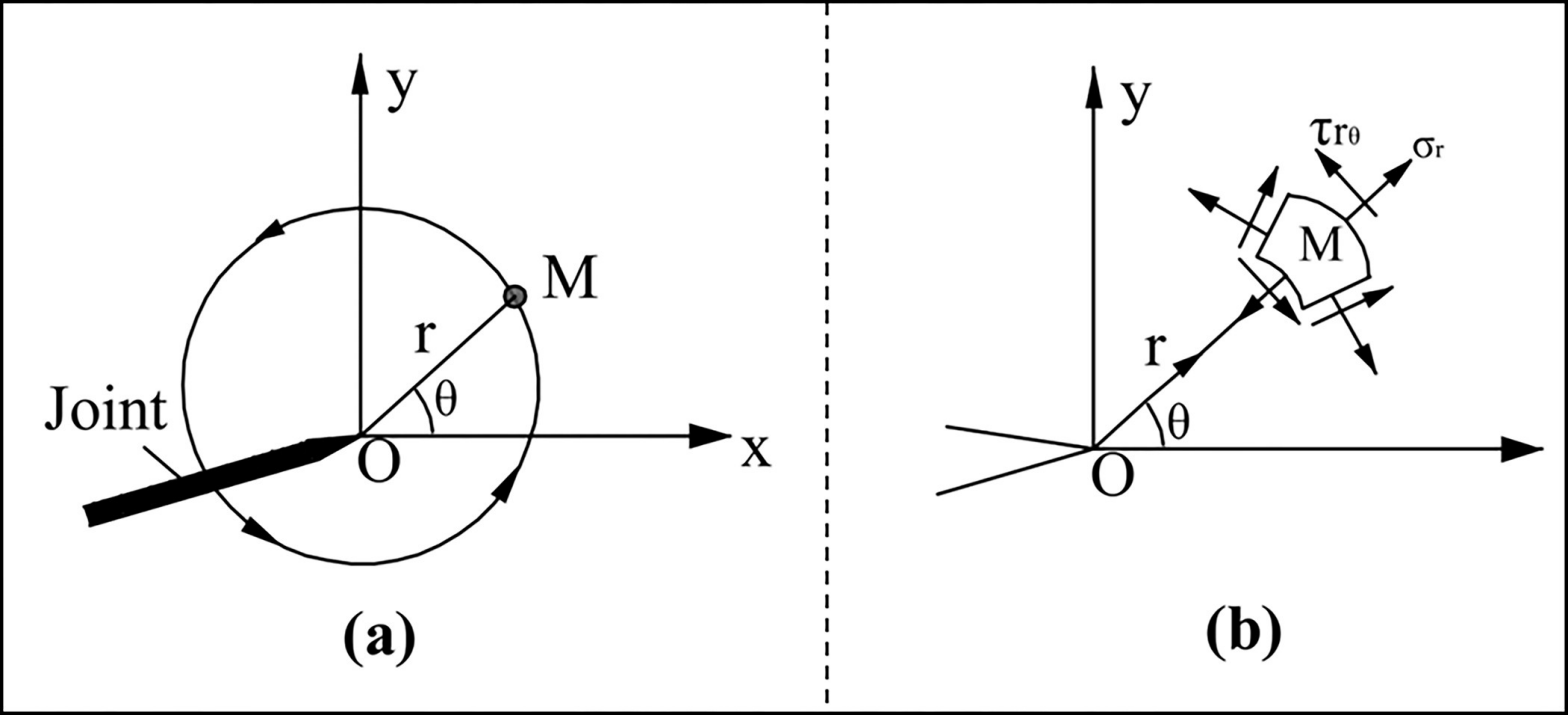
Polar coordinate representation of fracture tip: (a) J-integral loop around fracture tip (b) polar coordinate component of fracture stress.

The polar coordinate component of I-II composite fracture is shown in Fig 1. The linear elastic theory gives the stress expression near the tip in polar coordinates as follows [40]:

$$\sigma_{r}=\frac{1}{2\sqrt{2\pi r}}\left[k_{I}cos\frac{\theta }{2}(3-\cos\;\theta )+k_{II}sin\frac{\theta }{2}(3\cos\;\theta -1)\right]$$ (3)

$$\tau_{r\theta }=\frac{1}{2\sqrt{2\pi r}}\cos\frac{\theta }{2}[k_{I}\;\sin\;\theta +k_{II}(3\;\cos\;\theta -1)]$$ (4)

Where, *k*_*I*_ and *k*_*II*_ are type I and type II stress intensity factors on the fracture surface respectively; σ_r_ and τ_rθ_ are the radial stress and shear stress of the outer element of the crack tip in the polar coordinate system; *θ* stands for the angle from which the outer unit of the crack tip deviates from the original crack; *R* represents the distance between the element and the crack tip.

**Fig 2:**
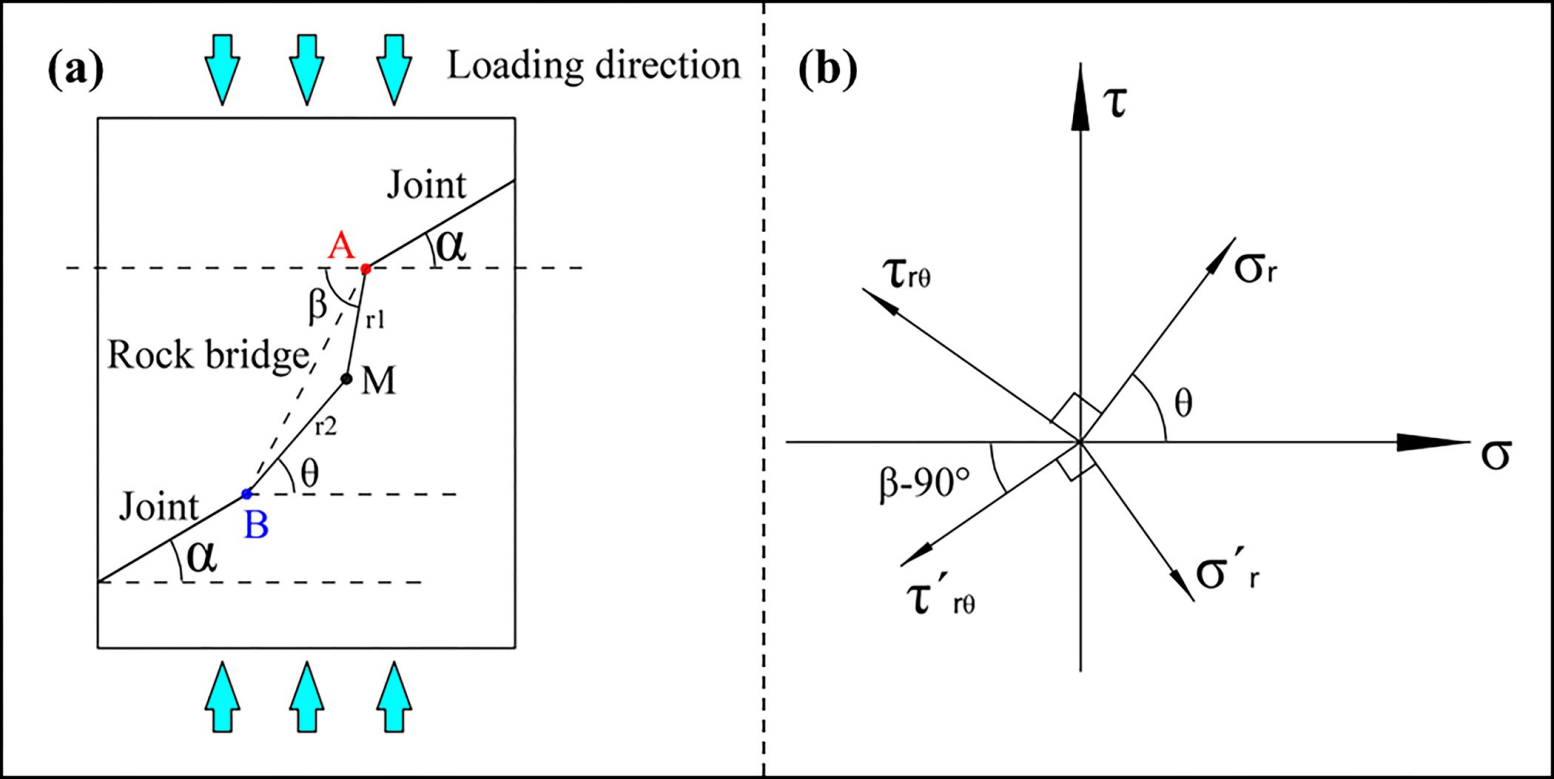
Non through parallel double joint model and stress analysis (a) non through parallel double joint model (b) horizontal stress analysis at point M.

In order to calculate the stress intensity factor, a stress analysis must first be performed on a double jointed rock sample. As shown in Fig 2, it is assumed that there are two non-penetrating parallel joints on the plane and not on the same straight line. The two nodal crack tips are A and B respectively, and the angle between the two nodal cracks and the horizontal direction is *α*. Take a point M at any position in the plane, and the linear distance between M and the tip of A joint fissure is *r*_1_. The linear distance between M and B joint crack tip is *r*_2_. The included angle between MA and horizontal direction is *β*. The included angle between MB and horizontal direction is *θ*.

Due to the symmetrical distribution of the double joints in the rock mass, the stress intensity factors generated by the two tips at point M are equal. In the polar coordinate system, the components outside the crack tip are simultaneously subjected to the radial stress σ_r_ and $\sigma_{r}^{‘}$ of both joints A and B:

$$\left\{ \begin{array}{c} \sigma_{r}=\frac{1}{2\sqrt{2\pi r_{1}}}[k_{I0}cos\frac{\theta }{2}(3-\cos\;\theta )+k_{II0}sin\frac{\theta }{2}(3\;\cos\;\theta -1)]\\ \sigma_{r}^{‘}=\frac{1}{2\sqrt{2\pi r_{2}}}[k_{I0}cos\frac{\beta }{2}(3-\cos\;\beta )+k_{II0}sin\frac{\beta }{2}(3\;\cos\;\beta -1)] \end{array}\right.$$ (5)

Simultaneously subjected to shear stress τ_rθ_ and $\tau_{r\theta }^{’}$ at A and B joints:

$$\left\{ \begin{array}{c} \tau_{r\theta }=\frac{1}{2\sqrt{2\pi r_{1}}}\cos\frac{\theta }{2}[k_{I0}\;\sin\;\theta +k_{II0}(3\;\cos\;\theta -1)]\\ \tau_{r\theta }^{’}=\frac{1}{2\sqrt{2\pi r_{2}}}\cos\frac{\beta }{2}[k_{I0}\;\sin\;\beta +k_{II0}(3\;\cos\;\beta -1)] \end{array}\right.$$ (6)

Coordinate systems in horizontal and vertical directions are established at point M for force analysis, as shown in Fig 2(B), and the equations are as follows:

$$\sigma_{r}\;\cos\;\theta +\sigma_{r}^{‘}\;\cos\left(180-\beta \right)=\tau_{r\theta }\;\cos(90-\theta )+\tau_{r\theta }^{’}\;\cos\left(\beta -90\right)$$ (7)

$$\sigma_{r}\;\sin\;\theta +\tau_{r\theta }\;\sin\left(90-\theta \right)=\sigma_{r}^{‘}\;\sin(180-\beta )+\tau_{r\theta }^{’}\;\sin\left(\beta -90\right)$$ (8)

By substituting Eq (5) and (6) into Eq (7) and (8), the expressions of k_I0_ and k_II0_ on M point position attribute M (θ,β,r_1_,r_2_) can be calculated.

When there are two parallel joints in the rock mass, the two joints will influence each other during uniaxial compression process. Assuming that the influencing factor is F(a,b,c,k,μ,β,B), then the stress intensity factor can be expressed as:

$$k_{I}=F{(a,b,c,k,\mu ,\beta ,B)k}_{I0},K_{II}=F{(a,b,c,K,\mu ,\beta ,B)K}_{II0}$$ (9)

Where, k_I0_ and k_II0_ represent stress intensity factors at the tip of a single non-penetrating joint respectively; *a* is the length of rock bridge; *b* is joint length; *c* is the spacing of parallel joint row; *K* is joint connectivity rate; *μ* is the friction coefficient of the structure plane; *β* is rock bridge dip angle; *B* is the width of specimen; F(a,b,c,k,μ,β,B) is the joint interaction coefficient.

Then, the stress intensity factor at the tip of the non-penetrating joint is:

$$k_{I}=M(\theta ,\beta ,r_{1},r_{2})F(a,b,c,k,\mu ,\beta ,B)=Y_{1}\sigma \sqrt{\pi b/2}$$ (10)

$$k_{II}=M(\theta ,\beta ,r_{1},r_{2})F(a,b,c,k,\mu ,\beta ,B)=Y_{II}\sigma \sqrt{\pi b/2}$$ (11)

In Eqs (10) and (11), Y_I_ and Y_II_ are dimensionless shape factors respectively, which can represent the interaction between joints.

For rectangular specimens, the joint surface area A = Bb, and Eq (10) and Eq (11) are substituted into Eq (1) to obtain the damage variable formula:

$$D=\frac{1}{1+\frac{2Bb\sqrt{\pi b/2}(1-v^{2})}{V}F(a,b,c,L,\mu ,\beta ,B)[(1-\frac{\sqrt{2\rho /b}}{2})\cos\;2\alpha +\sin\;2\alpha +\frac{\sqrt{2\rho /b}}{2}+1]}$$ (12)

Regarding the above calculation method, it can be found that the damage variables are affected by transverse stress, curvature radius of the joint tip and the deformation parameters of the joint surface (normal stiffness and tangential stiffness), etc. The influence of the transverse stress at the joint tip should be considered for the open-type non-penetrating joint. The length, dip angle and transverse stress of the joint can all affect the initiation path of rock mass.

## Experimental design and process

### Specimen design and preparation

In this work, uniaxial compression tests were carried out on non-through prefabricated double-jointed red sandstone in Junan County, Shandong Province. The samples are brick-red feldspathic sandstone and iron colluvium, and the main mineral composition is feldspar and quartz, with a small amount of montmorillonite, hematite and other minerals. The sandstone has a medium grain structure with a grain size of 0.1–0.35mm. It is a dense massive structure with relatively uniform grain size and macroscopically homogeneous. The average density of the sample is 2.235 g/cm^3^ and the average p-wave velocity is 4.649 m/s. In order to study the fracture damage characteristics of non-penetrating prefabricated double-jointed red sandstone under uniaxial compression, standard cuboid red sandstone with slenderness ratio of 2 and size of 50 mm×50 mm×100 mm was prepared. Specimens of red sandstone with two parallel joints and different geometrical state cracks were cut.

**Fig 3:**
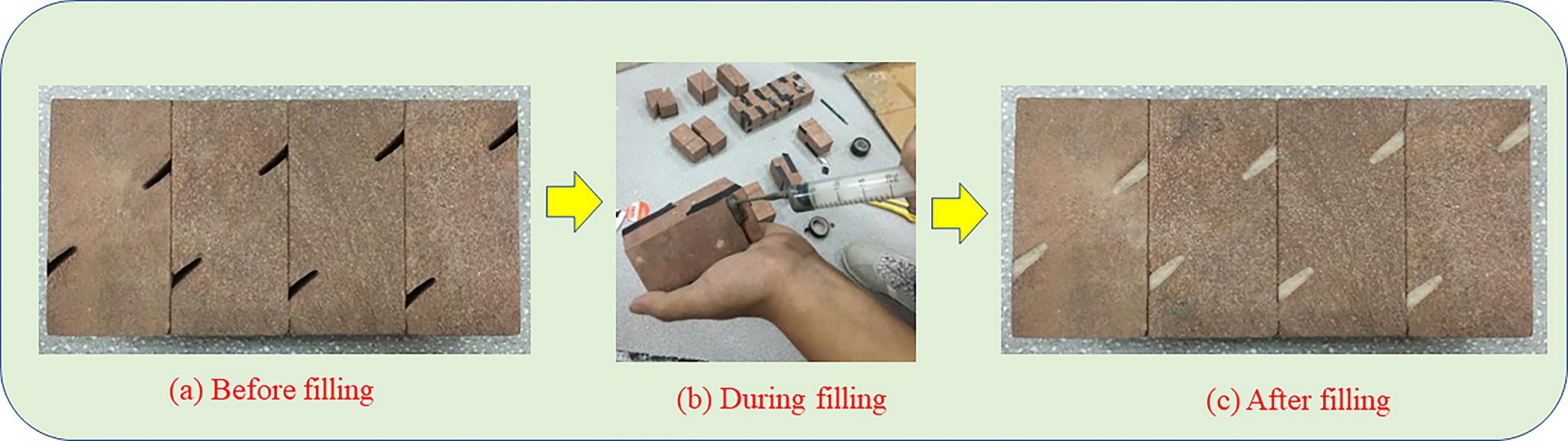
Specimen preparation process.

The specific steps for the fabrication of non-penetrating prefabricated double-jointed red sandstone specimens are as follows: (1) complete cuboid specimens were made; (2) rock samples under different geometric states are cut by joint cutting equipment, and the cutting thickness of rock sample joint is controlled at 2.5mm, as shown in Fig 3(A); (3) Joints were filled with cement mortar and sealed with adhesive tape as shown in Fig 3(B). After 48 h of complete solidification, the surface was polished. The flatness error of the four ends is less than 0.04 mm. Sample preparation was completed after screening, and the prepared samples were shown in Fig 3(C). The mechanical parameters of the complete rectangular samples were measured and the results are shown in Table 1.

**Table 1:** Physico-mechanical parameters of rock.

Name	Poisson’s ratio	Rock density *ρ*/g·cm^-3^	Tensile strength *σ*_t_/MPa	Uniaxial compressive strength *σ*_c_/MPa	Elastic modulus *E*_*T*_/GPa	Internal friction Angle/(°)
Red sandstone	0.375	2.623	9.69	112.9	34.3	31.5

### Experimental scheme

The damage modes of the specimens can be roughly categorized into two types: slippage damage along the joint plane and tensile failure through the joint plane. Both types of damage occur when the inclination angle is between 15° and 60°. Therefore, the uniaxial compression test is mainly carried out for red sandstone with the same dip angle of double joints and between 15° - 60°.

**Fig 4:**
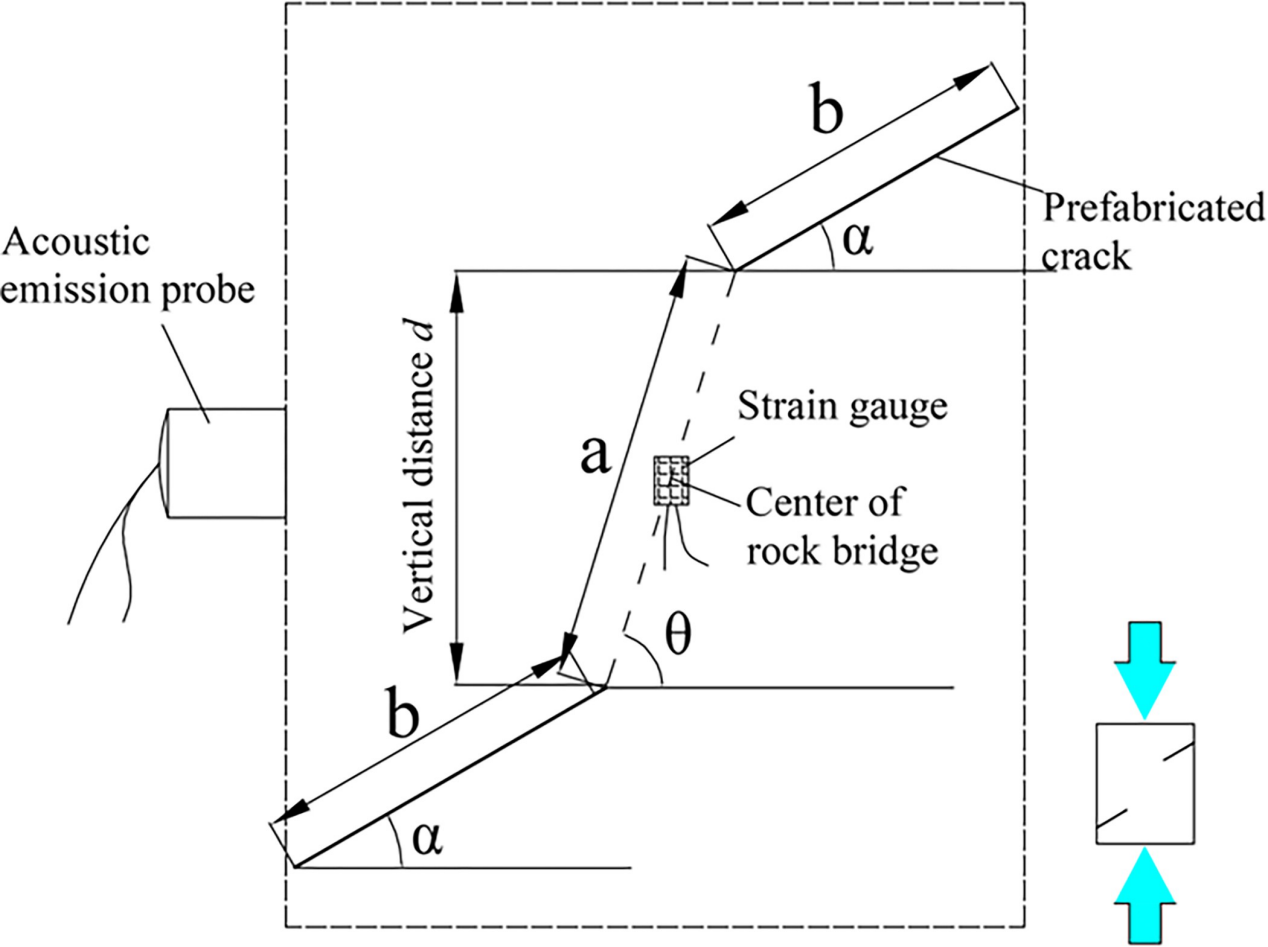
Geometric parameter distribution of specimens.

Here, in order to study the influence of different rock bridge lengths, connectivity and inclination angles on the fracture damage characteristics of red sandstone specimens with parallel double joints under uniaxial compression, four groups of tests were designed, with no less than four in each group. The specific experimental scheme is shown in Table 2, and the geometric parameter distribution of the corresponding specimen is shown in Fig 4.

**Table 2:** Geometric parameters of of rock specimens.

Number	Type	Remarks
Ⅰ	Full penetration- different joint dip angles α	α = 0°, 30°, 45°, 60°
ⅠⅠ	Double joint—different connectivity k	k = 0.3, 0.5, 0.7, 1
ⅠⅠⅠ	Double joint—different vertical distances d	d = 25mm, 35mm, 45mm, 55mm
ⅠV	Double joint—different joint dip angles α	α = 0°, 15°, 30°, 45°, 60°

**Fig 5:**
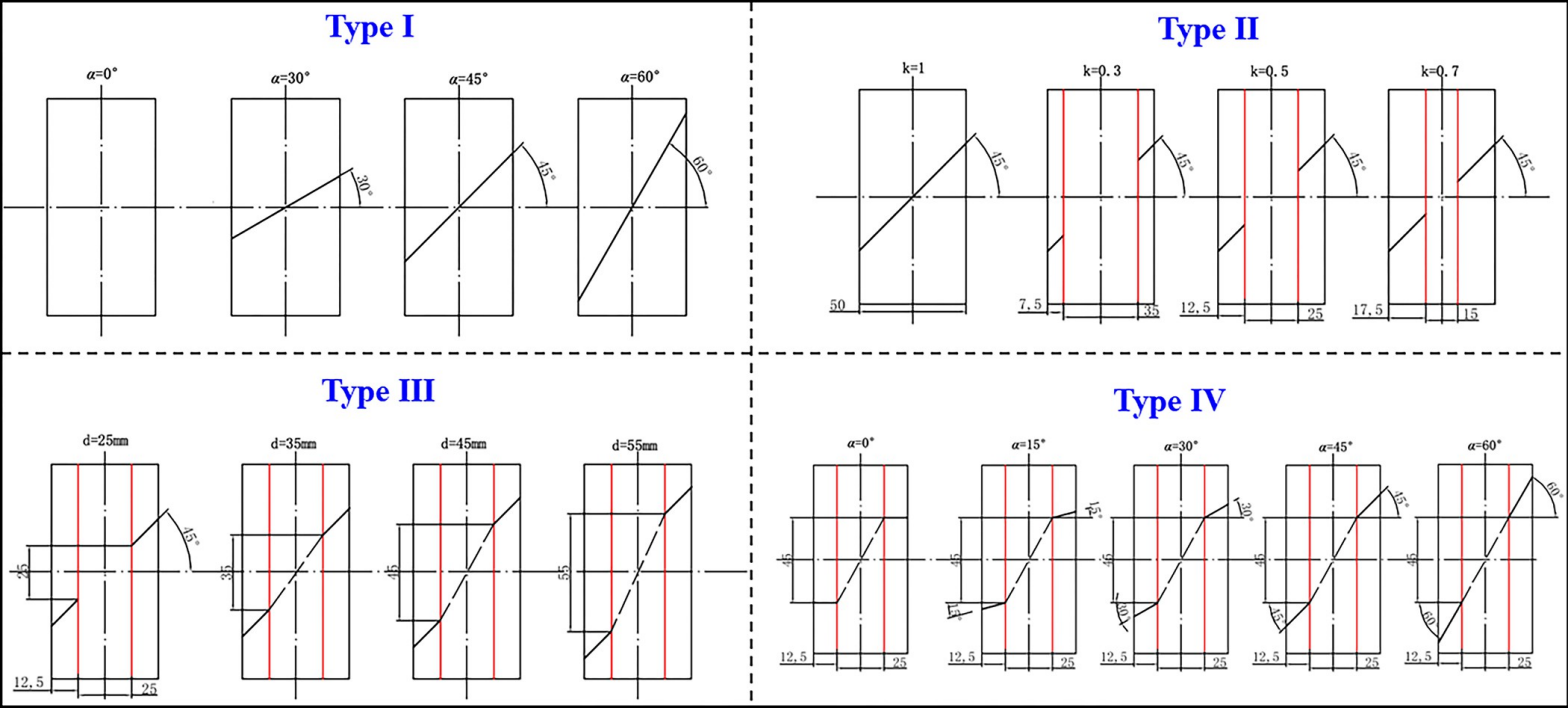
Specimen geometry.

Among them, type I is a full penetration specimen, and the main variable is the single joint inclination; the main variable of type II is the connectivity; the main variable of type III is the vertical distance between the tips in the joint. At this time, the length and inclination of the rock bridge are also changing at the same time; Type IV the main variable is the nodal inclination. In order to facilitate the understanding of our designed experimental program, the geometry of each group of experimental specimens is shown in Fig 5.

According to the connectivity formula:

$$k=\frac{S_{1}+S_{2}}{S_{1}+S_{2+}S_{3}}$$ (13)

Where: S_1_、S_2_ is the area of prefabricated double knots, and S_1_ = S_2_, S_3_ is the area of rock bridge.

Because the precast joint width of the cuboid specimen is equal to the width of the rock bridge, S_1_ = *bB*, S_3_ = *aB*, the prefabricated length *b* of two parallel joints is equal, and the length of rock bridge is *a*, so the formula is transformed into:

$$k=\frac{2b}{2b+a}$$ (14)

According to rock bridge length *a* and rock bridge inclination *β* and joint inclination *α*, the calculation formula of parallel joint row spacing is:

c=asin(β−α) (15)

### Experimental procedure

**Fig 6:**
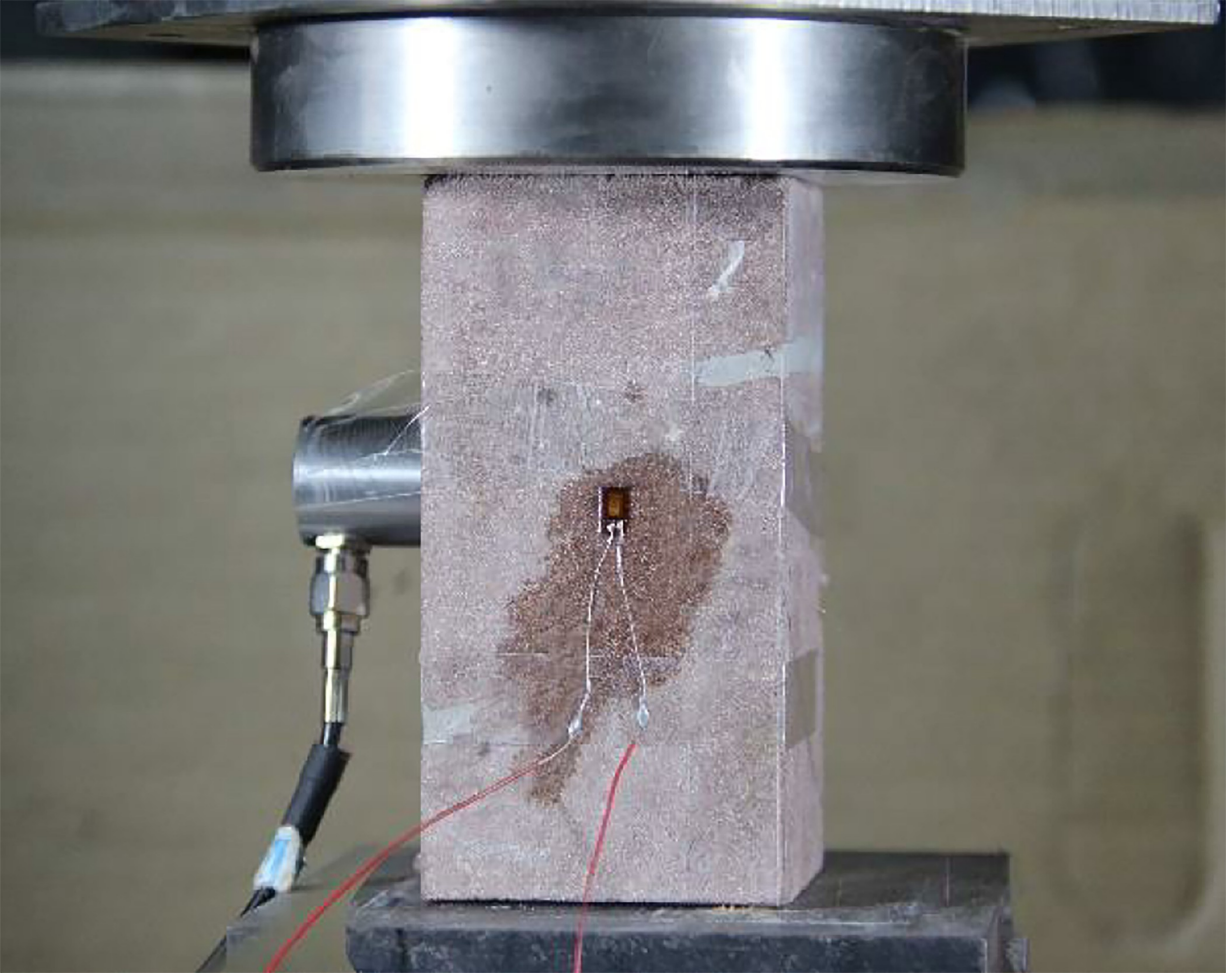
Loading and monitoring system for tests.

The test was carried out on AG-X250 universal testing machine, and the whole process stress-strain curves, deformation, strength and failure characteristics of samples with different rock bridge lengths, connectivity and inclination angles under uniaxial compression were deeply investigated. The maximum load of the testing machine is 250kN, the range of loading rate is 0.0005 mm/s~1000mm/s, the accuracy of speed control is ±0.1%, and the effective loading range is 595 mm. The test adopts the displacement control mode, the displacement rate is 0.005 mm/s, and the axial force is loaded to the destruction of the specimen. During this period, the test loading, stress and strain collection and photography should be carried out synchronously. In order to monitor the transverse strain law in the center of rock bridge, BFH120-3AA-Y3 strain gauge with sensitivity coefficient of 2.0±1% and resistance of 120 ohms was used. Before the test, the stress and strain sensor were set up in the middle of the line segment closest to the crack surface and weak surface of the specimen, as shown in Fig 6.

The acoustic emission monitoring and analyzing system of Misras series PCI-2 produced by American Physical Acoustics Corporation is used to monitor the acoustic emission signals generated by microfracture inside the red sandstone specimen to verify the method of characterization of fracture damage in rock bridges. Before the loading test, the sensor response should be measured and calibrated through the lead breaking test, and vaseline shall be applied on the test piece cushion block and the upper and lower end faces to reduce the end friction effect during the loading process. Fix the acoustic emission sensor. Ensure that the response amplitude exceeds 90dB, the floating threshold is 6 dB, the preamplifier gain is 40 dB, the sensor resonant frequency is 20 ~ 100 kHz, and the sampling frequency is 106 times/s.

## Results and analysis

### Stress characteristics of specimen

**Fig 7:**
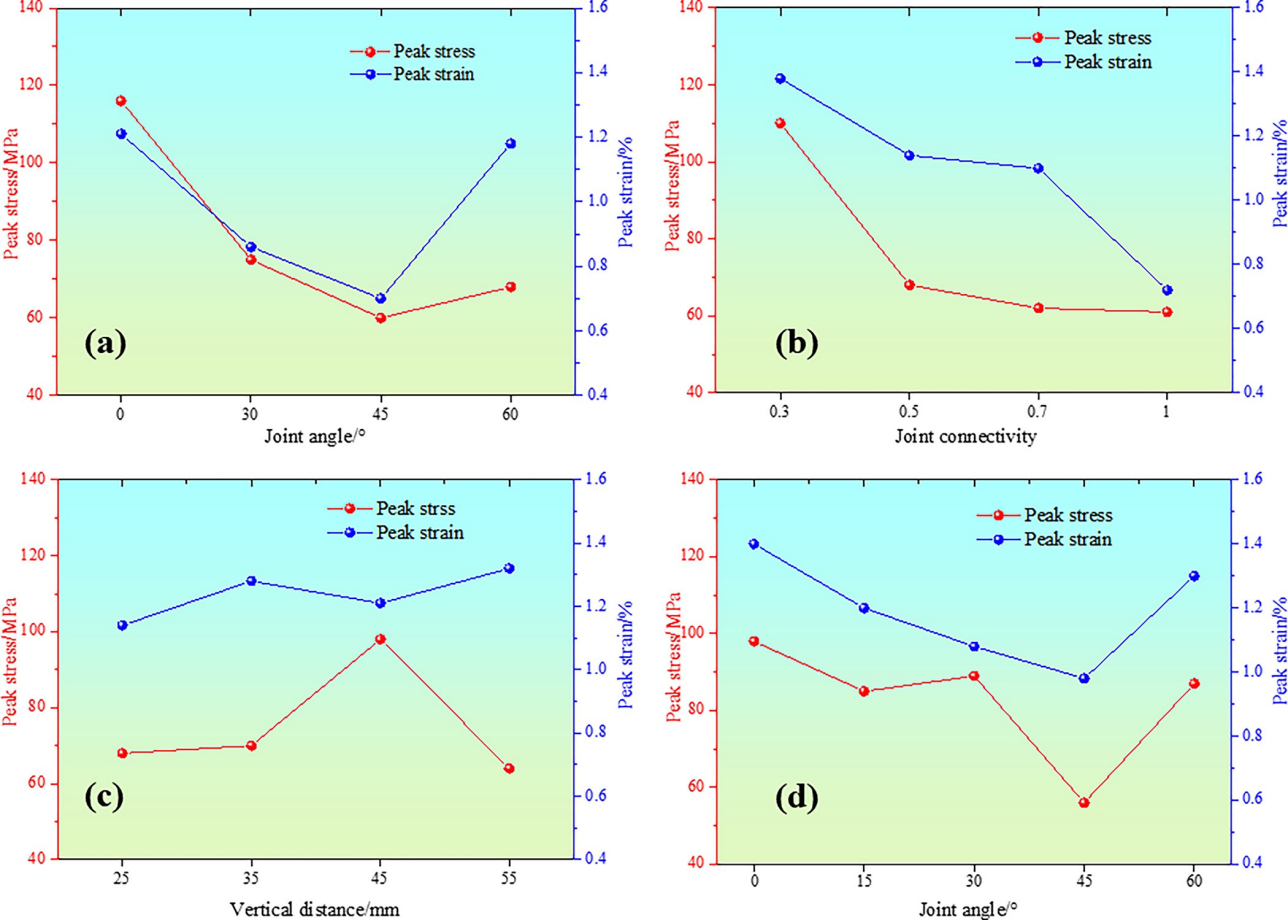
Peak stress-strain curves under different geometric parameters (a) Type I; (a) Type II; (c) Type III; (d) Type IV.

Uniaxial compression tests were performed on the four groups of specimens under the same conditions, and the stress-strain curves of the specimens were obtained, as shown in Fig 7. After reaching the peak stress, the specimens were obviously damaged.

As shown in Fig 7(A), the mechanical properties of a fully through specimen under uniaxial compression show certain regularity. With the increase of joint inclination angle, the peak stress and peak strain decrease first and then increase, and the minimum value occurs when the joint inclination angle is 45°. It indicates that the red sandstone specimens with 45° joint inclination angle are most vulnerable to vertical load failure and the failure time is the shortest. When the joint inclination angle is 0°, the peak strain is similar to that of the intact specimen, but the peak stress is still lower than 25MPa. Even if the joints are filled, the generation of filling joints will still lead to the weakening of peak strength. The peak stresses at nodal inclination angles of 30° and 60° are basically equal, but the peak strain of the latter increases significantly, indicating that the time to reach the peak stress is prolonged when the nodal inclination angle is greater than 45°.

As illustrated in Fig 7(B), the peak stress and strain decrease gradually with the increase of connectivity. When the connectivity is between 0.3 and 0.5, the peak stress decreases greatly, while when the connectivity is between 0.5 and 1, the peak stress decreases slightly. The peak strain is on the contrary. The peak stress decreases slightly between 0.3 and 0.5, and greatly between 0.5 and 1. This indicates that the peak stress has a greater effect on the length of the bridge during the initial stage of the reduction of the co-linear double-jointed rock bridge, while the peak strain has a greater effect on the length of the bridge as it approaches the connection stage.

As shown in Fig 7(C), the minimum values of peak stress and peak strain occur when the vertical distance of double nodes is 25 mm. It shows that the collinear joint is sensitive to axial compression load. When the vertical joint distance increases (the bridge length, bridge dip Angle and row spacing increase correspondingly), the peak stress and strain increases. It is suggested that increasing the vertical distance is helpful to restrain the joint tip. When the vertical distance between the two joints is 55mm, the peak stress decreases sharply. The results show that the distance between the joint tip and the upper and lower end faces affects the peak stress intensity limit under uniaxial action.

From Fig 7(D), it can be seen that the peak stress and peak strain decrease and then increase with the increase of the nodal inclination angle, and the peak stress is minimized at the nodal inclination angle of 45°. This is similar to the mechanical characteristics of the specimen with 45° joint dip Angle. When the nodal inclination angle is greater than 45°, the peak stress increases and the time to reach the ultimate stress is prolonged. The smaller the nodal angle, the smaller the peak stress.

### Rock bridge failure mode

**Fig 8:**
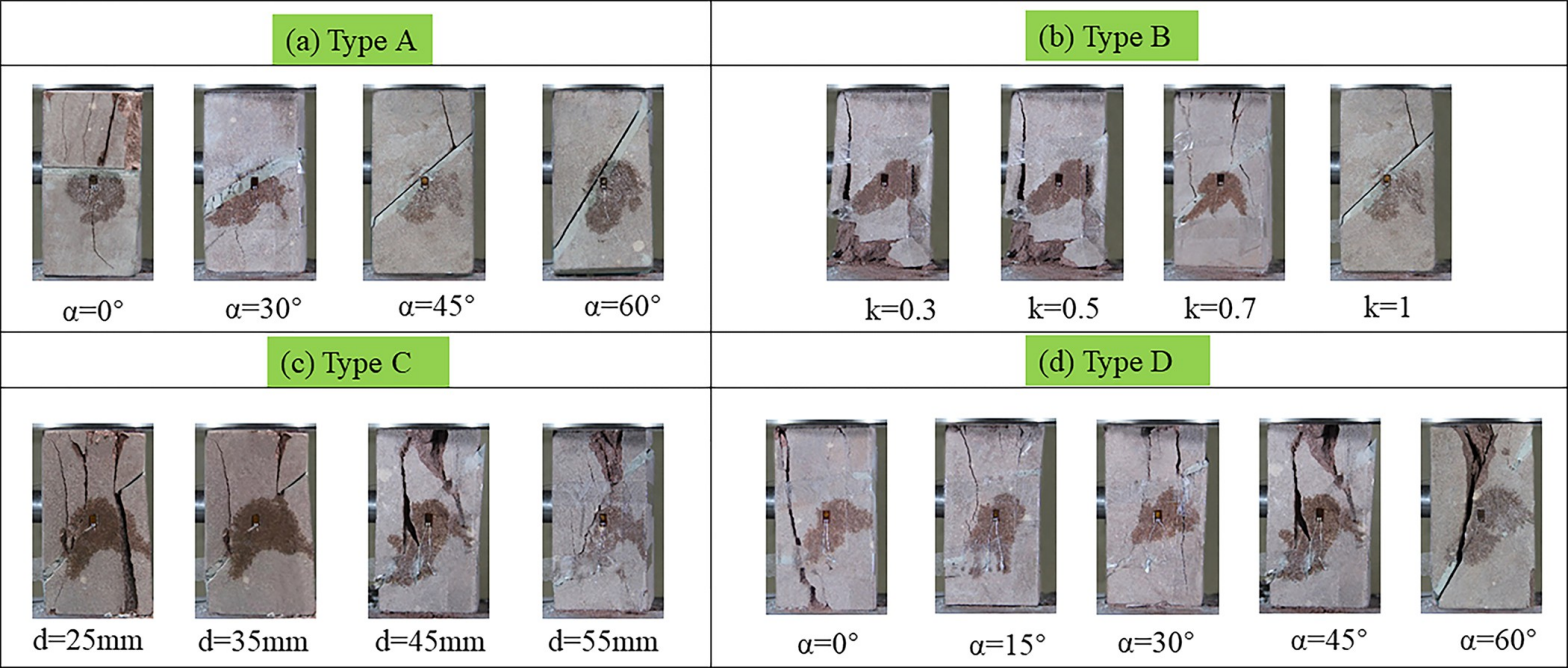
Failure modes of specimens under different joint parameters.

Due to the different structural modes of joints and fissures, rock damage and deformation show certain regularity. Crack morphology has significant influence on rock failure characteristics [41,42]. The damage patterns of specimens under uniaxial compression can be divided into four types: (i) Type A: slippage failure caused by the sprouting and expansion of a single jointed fracture. (ii) Type B: lap-through damage of adjacent tip cracks in prefabricated double joints. (iii) Type C: the fracture tip connects to the top of the rock showing overall brittle damage; (iv) Type D: the fracture tip penetrates to the rock bottom, showing overall brittle damage. The loading patterns of the four groups of specimens under different geometrical state parameters are shown in Fig 8.

As shown in Fig 8(A), the specimen as a whole mostly exhibits Type A failure when the inclination angle of the fully penetrating joint α is greater than 30°. When α is 45°, the damage fracture occurs in the upper segment of the filling fracture and spreads to the central position of the top, and the upper part of the fracture slides downward along the filling joint. This failure is defined as a special type of Type C failure. When α is 30°, the joint fissure occurs in the lower segment of the backfill and expands to the bottom center, which is defined as a special Type D failure. With the increase of the dip angle of the fully penetrating joint, the range of fracture development gradually decreases.

As shown in Fig 8(B), when the connectivity k is 0.3 and 0.5, the specimen produces Type C and Type D failure simultaneously. When k is 0.7, Type B, C and D failures occur simultaneously. When k is 1, Type A failure and special Type C failure occur. When k is 0.3, after peak strength, the left joint tip is connected to the upper end face and the right joint tip is connected to the lower end face. The propagation of joint tip is independent of the distance from joint to end face, but related to joint length and joint dip angle. When k is 0.5, more than one crack connecting to the upper end face occurs at the left joint tip after peak strength, and as k increases, one crack connecting to the upper end face occurs at the right joint tip. It shows that the deeper the joint tip, the more through-through cracks, and most of them are the upper through-through failure. When k is 0.7, the left and right sides of the nodules produce penetrating cracks above and below the upper face, which produces Type B damage. This indicates that the length of rock bridge is the key factor to produce Type B failure. In the case of double joints penetrating prefabricated fractures, the smaller the connectivity rate between the two fractures, the smaller the range of crack development.

As shown in Fig 8(C), when the vertical distance *d* of the parallel double joints is 25 mm, the two joints are in the same straight line, and two cracks through the upper end face are generated at the left joint tip after the peak strength. Simultaneously, downward expanding microcracks appeared, which were no longer connected after peak strength. The right joint tip produces a through-through fracture through the upper and lower surfaces, that is, the left joint is Type C failure, and the right joint is C and D failure. This indicates that when α is 45°, the joint tip cracks and expands along the tip direction under uniaxial compression, while the joint tip near the loaded end (right joint) is more prone to through-cracking. When *d* is 35mm, the downward extending micro-fissure disappears, and the penetrating upper end face fissure generated by the right joint tip shifts to the right, indicating that the penetrating is more "urgent". When *d* is 45mm, the left joint produces a more "specialized" C-type failure with only one through fracture. The type D failure of the right joint disappears, and the joint tip produces a through-through fracture connecting the right end face, which also reflects the "urgency" of the penetration, and the opening of the penetration is located in the middle of the distance between the opening of the right joint and the bottom face. When *d* is 55mm, the specimen obviously produces Type B failure, indicating that the rock bridge angle is one of the key factors for the connection of double joints. In the case of double joints penetrating prefabricated fractures, the smaller the vertical distance between the two fractures, the smaller the range of crack development, and the more serious the sample damage.

The failure process and crack propagation patterns in the rock-like material specimens were affected by changes in cracks’ angles and their configurations [43]. As shown in Fig 8(D), when the joint inclination Angle α is 0°, Type C and D damages appear at the left joint tip, and the two damages are connected with each other. The upper penetration crack is located at the left edge of the upper end face, and the right joint tip produces Type C failure micro-crack. When α is 15°, only the left joint tip shows Type C damage, while the upper penetration fracture is displaced to the right side, and the Type C damage microfracture is more pronounced at the right joint tip. When α is 30°, the left joint tip still produces only Type C failure, while the right joint tip produces obvious Type C failure through crack and downward spreading micro-crack, which is not connected after peak strength. When α is 45°, only the left articular tip underwent Type C destruction, and the superior through fracture continued to move to the right. The right joint tip did not produce Type D failure, but produced a through fracture connecting the right end face, reflecting the "urgency" the through fracture, and the opening of the through fracture was located at the middle of the distance between the opening of the right joint and the bottom face. In addition, the center of the bridge produces microfractures perpendicular to both the top and the bottom, which do not connect after peak strength. When α is 60°, only Type C failure occurs at the left and right joint tips. It indicates that the existence of joint tip poses a certain threat to the fracture damage near the tip. In the case of double joints penetrating prefabricated fractures, the larger the joint dip angle between the two fractures, the larger the range of crack development, and the more serious the sample damage.

### Lateral strain

**Fig 9:**
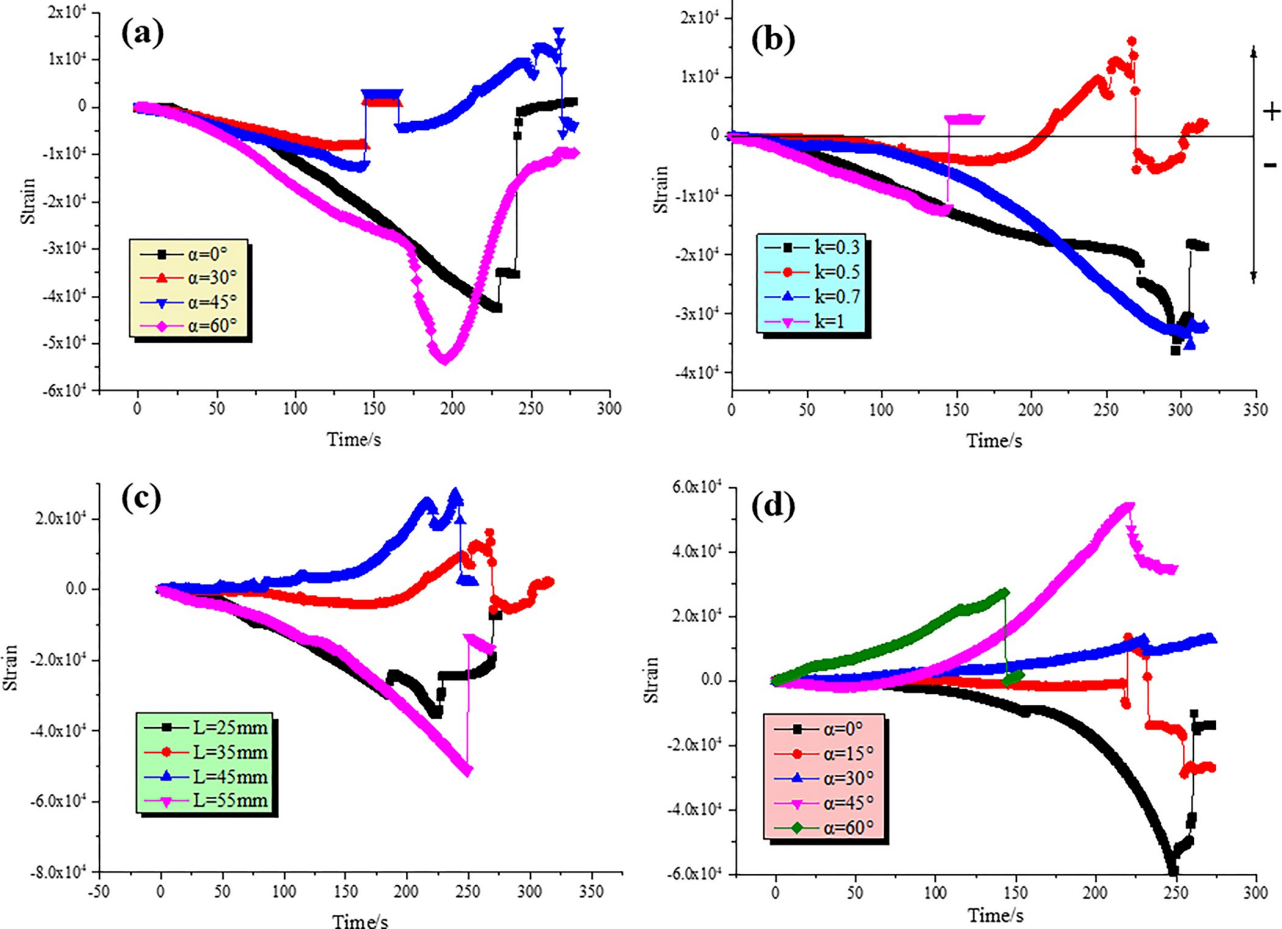
Transverse displacement curve of rock bridge center under different geometric parameters (a) Type A; (a) Type B; (c) Type C; (d) Type D.

The lateral strain data at the center of the rock bridge under four groups of tests were recorded. The lateral shift curves of the rock bridge center under different geometric parameters are shown in Fig 9. It can be seen from Fig 9(A) that the maximum strain is positive and the center of rock bridge moves to the right only when the nodal inclination angle is 45°. The maximum strain of other joint dip angles is negative. As shown in Fig 9(B), only when the connectivity rate k is 0.5, the maximum strain is positive and the center of rock bridge moves to the right. When the connectivity k is 0.5 and 0.7, the maximum strain is negative and the center of the bridge moves to the left. Both values of maximum strain are essentially the same, indicating that connectivity is independent of lateral displacement of the bridge center, but the concavity of strain curvature is opposite. When k is 0.3, the lateral strain values change from sharp to slow. When k is 0.7, the lateral strain value changes from slow to sharp. The results show that an increase in connectivity affects the rate of change of transverse strain at the center of the rock bridge for the same strain time and peak strain variable. When the connection rate k is 1, the total strain time is shortened from 300 s to 148 s, and the peak strain variable is reduced from 36000 to 12000 strains. The results show that the transverse displacement of the filled single joint is more stable than that of the parallel double joint.

As shown in Fig 9(C), when the vertical distances of the parallel double joints are 35mm and 45mm respectively, the maximum strain is positive and the center of the rock bridge moves to the right. When the vertical distance is 55mm, the strain is negative and the center of the bridge moves to the left. Combined with Fig 8, it can be seen that the damage mode of red sandstone is related to the lateral displacement of the rock bridge center. When the vertical joint distance is 35mm, the right joint produces Type C and Type D failure simultaneously. When the vertical distance of the joints is 45 mm, the right joint undergoes Type C damage and cracks appear on the right end face. The similarity between the two is that the right part of the specimen is partially separated, resulting in the transverse strain in the center of the rock bridge developing to the right. At a vertical joint distance of 45 mm, the "urgency" of penetration shortens the time to reach maximum strain. When the vertical joint distance is 55mm, the lateral displacement of the bridge center reaches -50000 strain. The connection method of the rock bridge has a great influence on the lateral displacement of the rock bridge center.

As shown in Fig 9(D), when the joint inclination angle is 0°, the left lateral displacement of the rock bridge center reaches 56000 strains, and both Type C and Type D damage occurs on the left side of the nodal. This damage pattern is the same as that of the specimen with a vertical joint distance of 35 mm, but in the opposite direction. The transverse strain in the center of rock bridge develops to the left. When the nodal inclination angle is 15°, both left and right sides show c-type damage trend, and the transverse displacement reaches a certain degree of "equilibrium". When the inclination angle of the joint is 30°, the right side of the joint shows Type C and D failure trend, and the transverse strain in the center of rock bridge begins to develop to the right, but the strain variable is not large. When the nodal inclination angle is 45°, the transverse strain in the center of the bridge develops to the right to 50,000 strains. The Type C and Type D damage produced by the left joint lags behind the right end face penetration damage produced by the right joint in time, thus producing positive strains. When the nodal inclination angle is 60°, the lateral displacement of the bridge center reaches -28000 strain, and the time to reach the maximum strain is shortened to 148 s. As a result, the conclusion that the transverse displacement of the rock bridge affects the lateral displacement of the rock bridge center is verified by Fig 8(D), which shows that the transverse movement of the rock bridge affects the lateral displacement of the rock bridge center at a vertical distance of 55 mm.

### Acoustic emission characteristics

**Fig 10:**
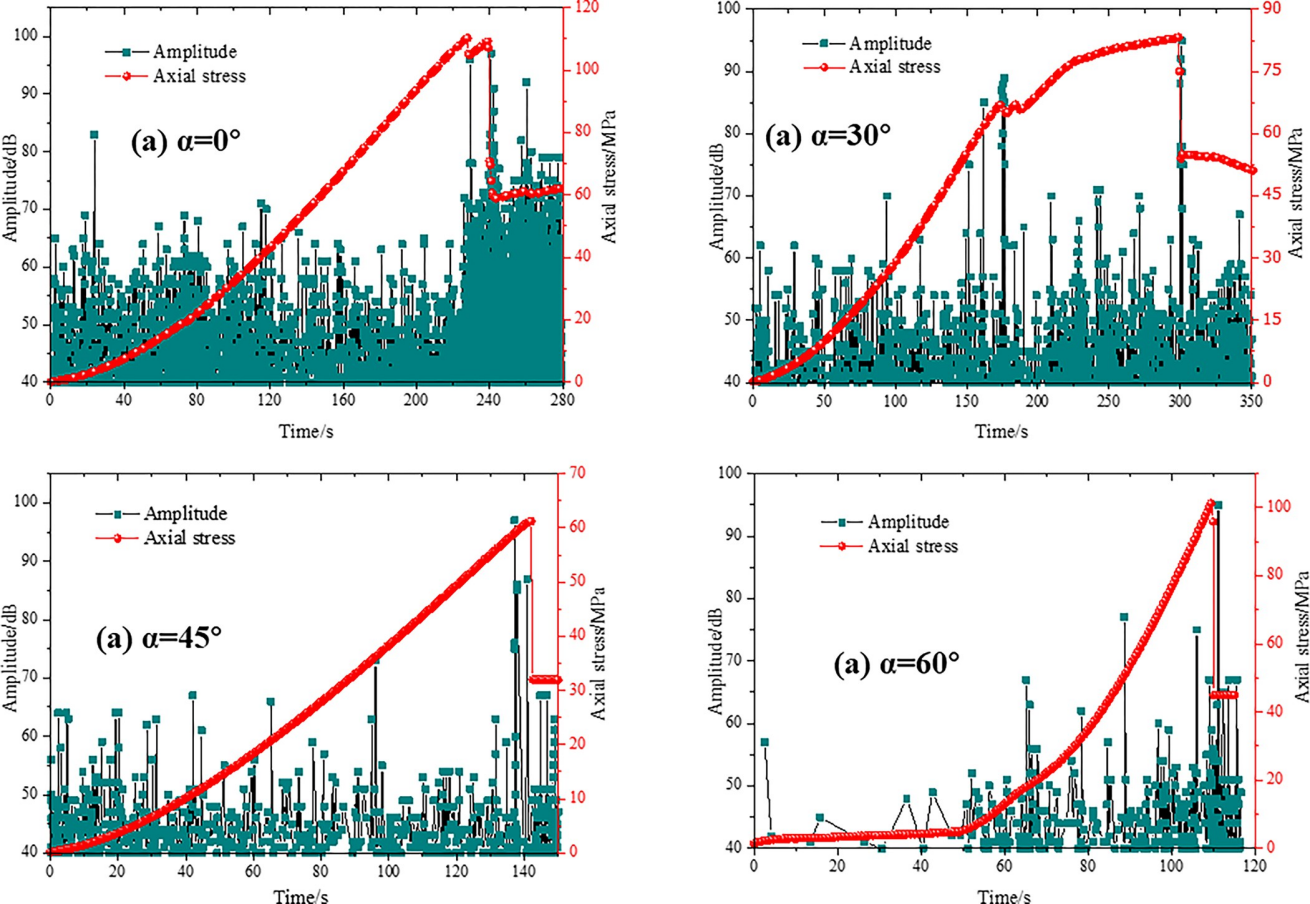
Acoustic emission characteristics of type I test.

**Fig 11:**
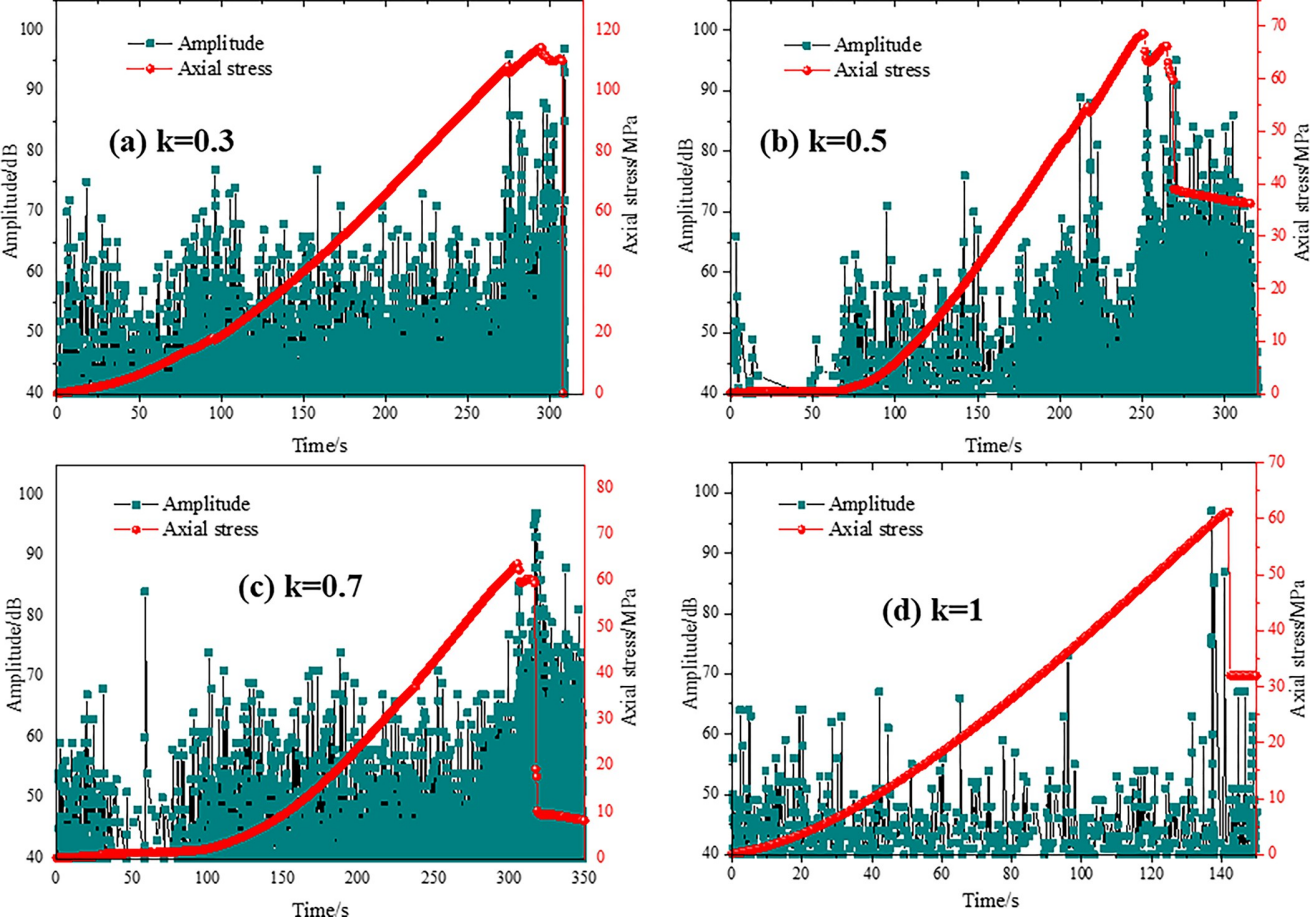
Acoustic emission characteristics of type II test.

**Fig 12:**
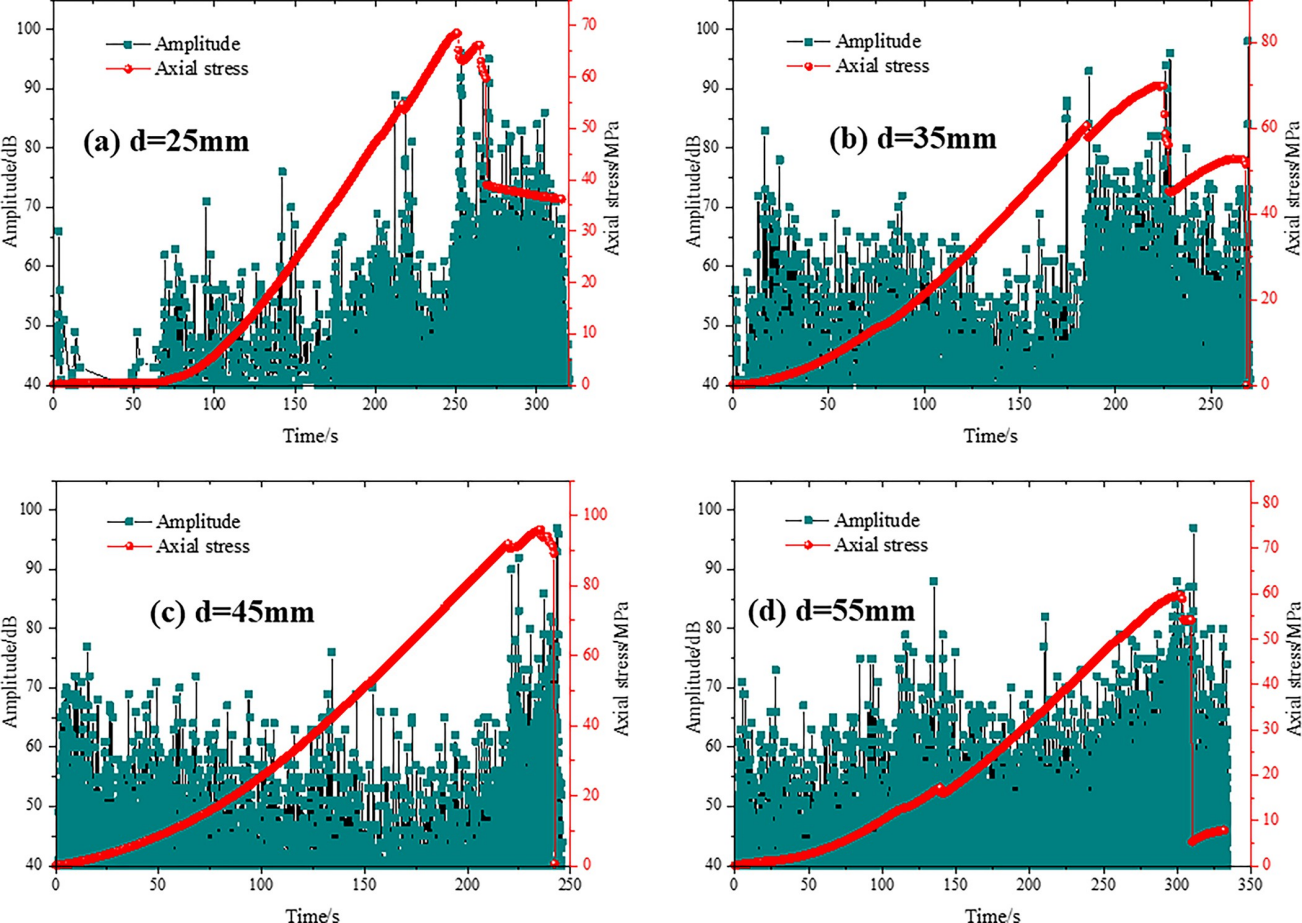
Acoustic emission characteristics of type III test.

**Fig 13:**
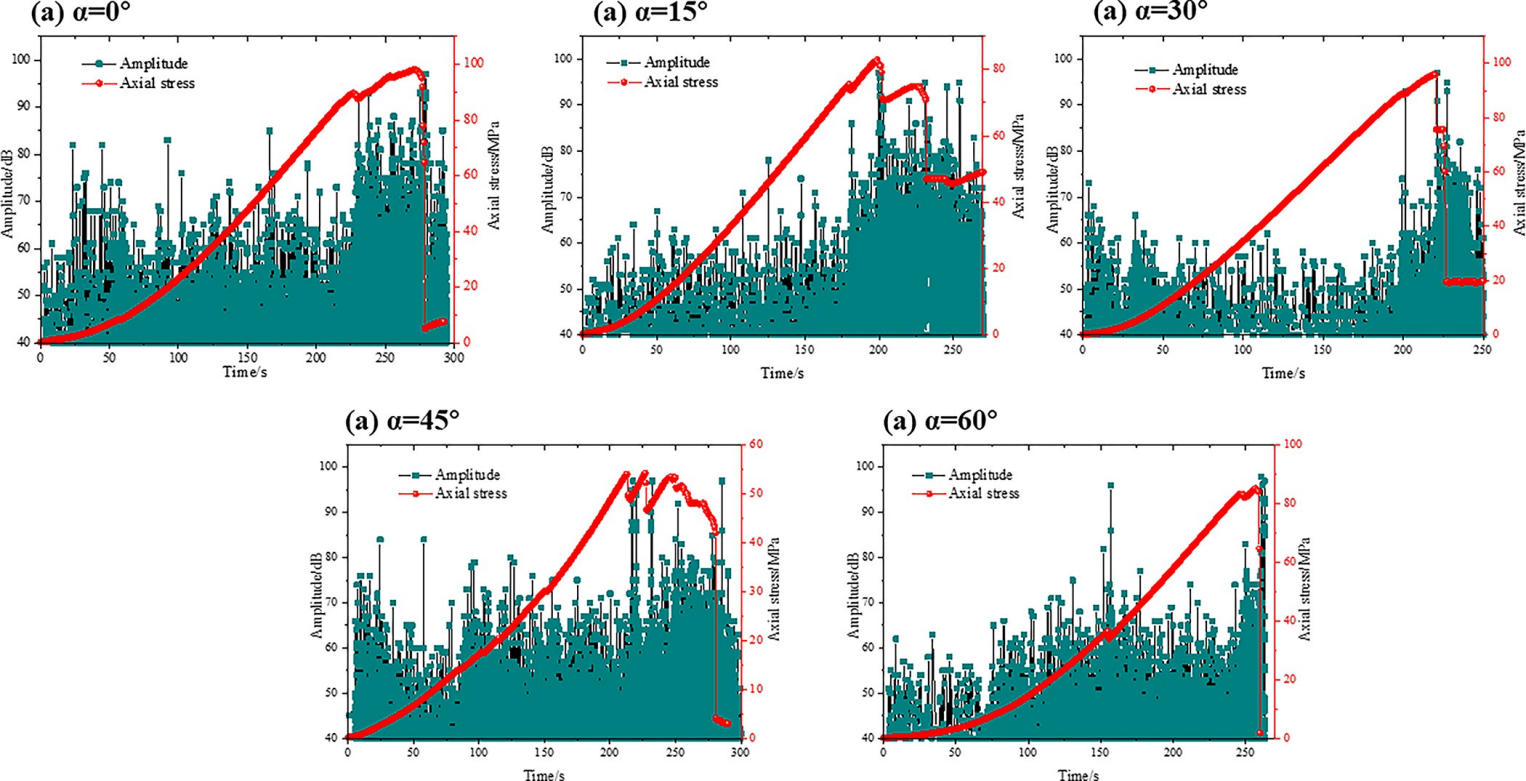
Acoustic emission characteristics of type IV test.

Under uniaxial pressure, the acoustic emission (AE) characteristics of specimens with different prefabricated crack lengths varied with time as shown in Figs 10–13. The axial stress-time-amplitude curves of each specimen were analyzed, and it was found that the axial stress of the four test groups presented "stepwise" changes, and the amplitude increased significantly at the stepwise changes. When the specimens reached the yield limit, the amplitude decreased sharply.

As shown in Fig 10, there are two abrupt changes and two step changes in stress when the inclination of the through joint is less than 45°. When the nodal inclination angle is greater than 45°, the step change occurs only once, which is presumed to be related to the overall shear damage. In the stress—free stage, the energy rate is low and does not change greatly. This indicates that cracks and microcracks are basically absent in the interior. When a "step" change in stress values occurs, the number of acoustic emission (AE) events increases, macroscopic cracks appear on the surface, and wing cracks or tensile cracks associated with prefabricated crack tips are observed. As the load increased to the peak strength, AE event distribution began to gather and various types of cracks appeared. Subsequently, the stress drops linearly, the strength does not rebound, and the specimen cracked as a whole.

As shown in Fig 11, the peak stress decreases gradually when the connectivity of specimen K gradually increases. Before the sudden change, the peak stress amplitude fluctuates within 70, and the maximum value appears in the last step change, and the value is basically the same, about 95. It indicates that the destruction of the red sandstone specimen is the main cause of the sudden change. When the connectivity rate is less than 0.7, the stress change is divided into two steps. Immediately after the first abrupt change, the amplitude increases sharply and the peak stress has not yet appeared. When the specimen reached the peak strength, the stress changed again, the amplitude fluctuated violently, and then the stress decreased linearly, the strength did not rebound, and the specimen shows an overall fracture phenomenon. When the connectivity is 1, the specimen reaches the peak strength and the maximum amplitude.

As shown in Fig 12, the axial stress has undergone at least two abrupt changes. It can be seen that under uniaxial compression, fracture propagation of red sandstone specimens can be clearly divided into three stages: I-initial compact-elastic stage, II-initial crack evolution stage and III-macroscopic crack failure stage. This is also consistent with the results of literature [9,44]. When the vertical distance is 55mm, the occurrence time of stage II is significantly advanced, indicating that the distance between the joint tip and the end face will affect the occurrence time of Type C and D failure. As the vertical distance increases, the distance between the joint tip and the end face becomes smaller, and the amplitude of the first mutation decreases from 99 to 87, indicating that the earlier the failure of Type C and Type D occurs, the smaller the acoustic emission characteristics are.

As shown in Fig 13, the initial dense-elastic stage, the initial crack evolution stage, and the macroscopic crack damage stage all appeared, and the axial stress and amplitude changed twice. When the inclination angle of parallel double joint is 60°, the occurrence time of stage II damage is significantly earlier, indicating that the occurrence time of Type C and Type D failure is not only related to the distance between the joint tip and end, but also related to the joint inclination angle. The smaller the angle between inclination angle and stress direction, the shorter the breaking time.

In conclusion, the red sandstone specimens are highly homogeneous and elastically brittle. The crack initiation and failure of the specimen are characterized by fast expansion, high energy rate and large amplitude. The red sandstone specimen with parallel double joints is prone to produce Type C and Type D failure during fracture initiation, which inevitably generates a step wave and corresponds to the fluctuation of acoustic emission amplitude. During the loading process, stress concentration occurs at the prefabricated parallel double joint tip, and the energy is accumulated and released, forming the peak of the acoustic emission signal. At the moment of fracture initiation, the load carrying capacity of the specimen is temporarily reduced and the axial stress curve shows a step change.

## Conclusions

In this paper, prefabricated joints were set up in red sandstone and uniaxial compression and acoustic emission tests were carried out to analyze the effects of different joint dip angles, rock bridge dip angles and connectivity on the strength and failure modes of the specimens. The main conclusions are as follows:

1. Under uniaxial compression, the peak stress and strain of the fully through-through specimen with a joint inclination Angle of 45° is the minimum. When the joint inclination Angle is greater than 45°, the time to reach the peak stress becomes longer. The peak stress and strain decrease with the increase of the connectivity.
2. Joint dip Angle, rock bridge dip Angle and connectivity synergistically affect joint tip through-through failure mode. The crack propagation of joint tip is independent of the distance from joint to end face, but related to joint length and joint dip Angle. The joint tip will generate cracks in the corresponding direction and continue to expand, and the joint tip near the load will generate through cracks ahead of the parallel cracks far away in time. The closer the joint tip is to the load, the more likely the end face through crack occurs. The dip Angle of rock bridge is constant.
3. The connectivity rate has nothing to do with the lateral displacement of the rock bridge center. Under the same strain time and peak strain variables, the increase of connectivity will affect the change rate of transverse strain in the center of rock bridge. After filling, the transverse displacement of fully through single joint is more stable than that of the red sandstone specimen with parallel double joints. The coalesce of rock bridge will greatly affect the central transverse displacement of rock bridge, and the coalesce failure mode of joint tip will affect the central transverse displacement of rock bridge, and the displacement is always close to the first part of the rock bridge.
4. The fracture propagation stress of red sandstone specimen has two abrupt changes and two step-like changes. The distance between the joint tip and the end face affects the occurrence time of Type C and Type D failure. The earlier the occurrence of Type C and Type D failure, the smaller the acoustic emission characteristics. The occurrence time of Type C and Type D failure is related not only to the joint tip and end distance, but also to the joint inclination angle.

The research content is expected to provide a certain theoretical basis for ensuring the stability of underground engineering mining. Admittedly, the samples selected in this paper are not extensive enough, and the findings obtained are relatively special. More extensive experimental samples need to be carried out in the future to consolidate the conclusions.

## Supporting information

S1 Data (PDF)
