# Supplementary material for: Investigation on uniaxial compression and fracture damage mode of prefabricated parallel double-jointed red sandstone
Source: PLoS One. 2024 Jun 17;19(6):e0305565. doi: 10.1371/journal.pone.0305565 (PMC11182494; doi:10.1371/journal.pone.0305565)
Supplement: S1 Data — (PDF) [file pone.0305565.s001.pdf]

Minimal data

Acoustic emission characteristics of type I test (α=0°)

Time/s	Amplitude/dB
1.79283	43
1.94104	58
1.96105	40
2.04705	52
2.09969	41
2.128	41
2.17791	40
2.24015	41
2.24785	57
2.26258	46
2.34094	48
2.38897	44
2.39518	65
2.402	42
2.40471	47
2.50953	40
2.53914	41
2.64004	51
2.66738	53
2.79955	49
2.8728	42
2.88766	40
3.02462	47
3.06755	44
3.13244	50
3.13453	40
3.578	49
3.81213	40
4.26756	45
4.53645	49
4.54742	41
4.76999	47
4.86831	43
4.9597	46
5.02561	41
…..	……

278.89801	57
278.90119	43
278.91011	54
278.91563	48
278.91863	42
278.92721	42
278.92975	45
278.9379	48
278.94215	40
278.94489	50
278.94744	45
278.95065	43
278.95286	47
278.95656	42

Acoustic emission characteristics of type I test (α=30°)

Time/s	Amplitude/dB
1.0196	32
1.42078	42
1.48165	37
1.54297	42
1.60124	32
1.6041	30
1.75688	33
1.80309	44
1.86006	35
1.94098	36
1.96502	52
1.97646	34
2.00673	30
2.03499	37
2.08245	36
2.14088	30
2.23452	30
2.28166	48
2.36815	46
2.40762	42
2.40994	34
2.60562	36
2.78223	31
2.89197	35

2.98857	35
3.03976	30
3.06727	39
3.29626	39
3.40228	41
3.41292	34
3.42848	39
3.43467	52
3.4386	31
3.57025	30
3.57357	30
…..	……
368.62205	36
368.67766	39
368.79916	47
368.82744	38
368.85399	41
368.89349	38
368.91334	39
369.02533	30
369.02765	43
369.03529	44
369.42975	32
369.45951	31
369.47126	38
369.49738	33

Acoustic emission characteristics of type I test (α=45°)

Time/s	Amplitude/dB
0.05335	49
0.06884	41
0.20138	56
0.22046	40
0.27755	46
0.41372	50
0.45333	45
0.47943	45
0.54934	40
0.62101	45
0.7735	41
0.81717	48
0.95282	44

1.06355	44
1.08361	41
1.15587	49
1.16329	45
1.24366	42
1.26569	45
1.37788	49
1.41586	41
1.53292	40
1.67705	41
1.73819	42
1.98183	43
2.16705	43
2.19256	47
2.20057	40
2.21131	44
2.26508	49
2.27005	44
2.30564	64
2.50479	46
2.58832	42
2.85164	40
…..	……
147.1	51
147.2	41
147.3	47
147.4	40
147.5	41
147.6	41
147.7	49
147.8	46
147.9	41
148	43
148.1	45
148.2	47
148.3	49
148.4	51

Acoustic emission characteristics of type I test (α=60°)

Time/s	Amplitude/dB
2.55207	57
4.14867	42
13.63163	41
15.80989	45
26.45747	41
30.59663	40
32.44376	42
36.57336	48
40.47494	40
42.75308	49
47.48203	42
49.48646	42
50.89681	47
51.39308	40
52.13117	52
52.23429	42
52.27218	43
53.23295	49
53.73396	48
53.96719	46
54.15538	45
54.59322	45
55.11774	41
55.2058	41
55.20772	40
55.21281	40
55.21524	40
55.45898	43
56.04684	40
56.135	41
56.13762	40
56.26433	45
56.38061	50
57.09634	47
57.98965	41
…..	……
114.3	40
114.4	41
114.5	41
114.6	41

114.7	47
114.8	40
114.9	41
115	41
115.1	40
115.2	46
115.3	40
115.4	51
115.5	40
115.6	44
